# Supplementary figures and images for: 5-Methylindole kills various bacterial pathogens and potentiates aminoglycoside against methicillin-resistant Staphylococcus aureus
Source: PeerJ. 2022 Sep 14;10:e14010. doi: 10.7717/peerj.14010 (PMC9482361; doi:10.7717/peerj.14010)

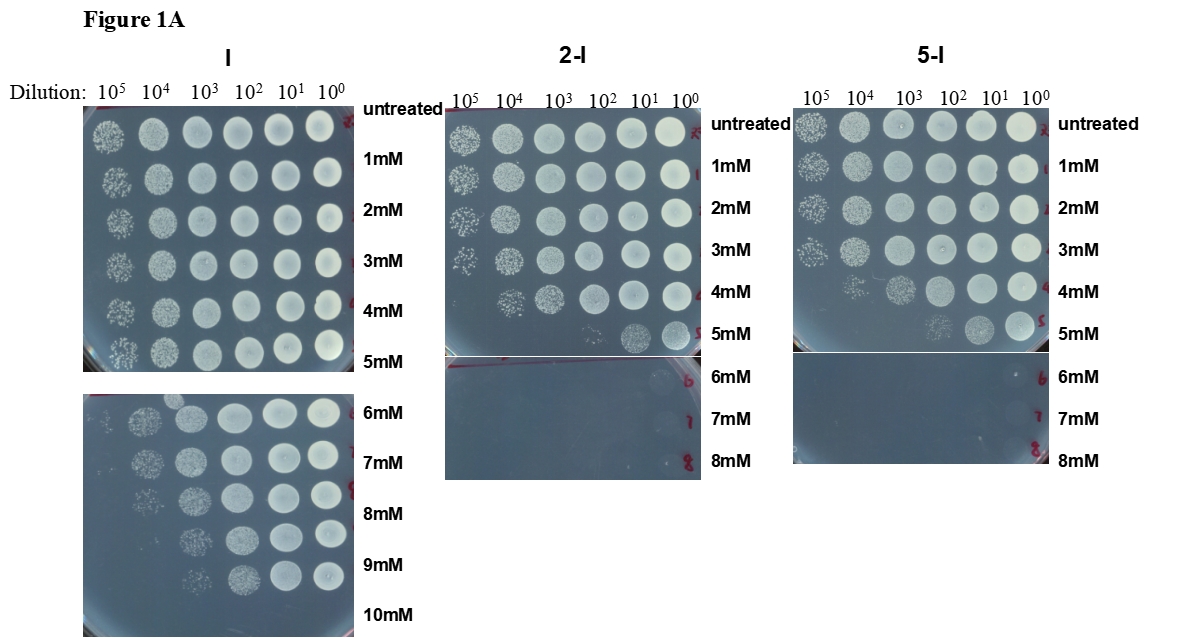


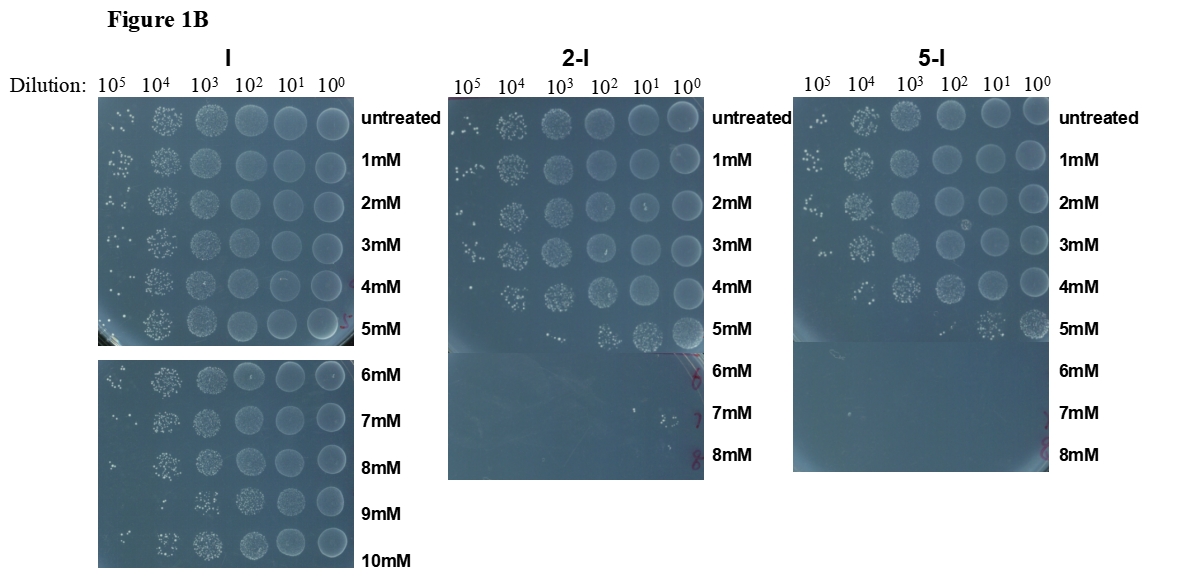


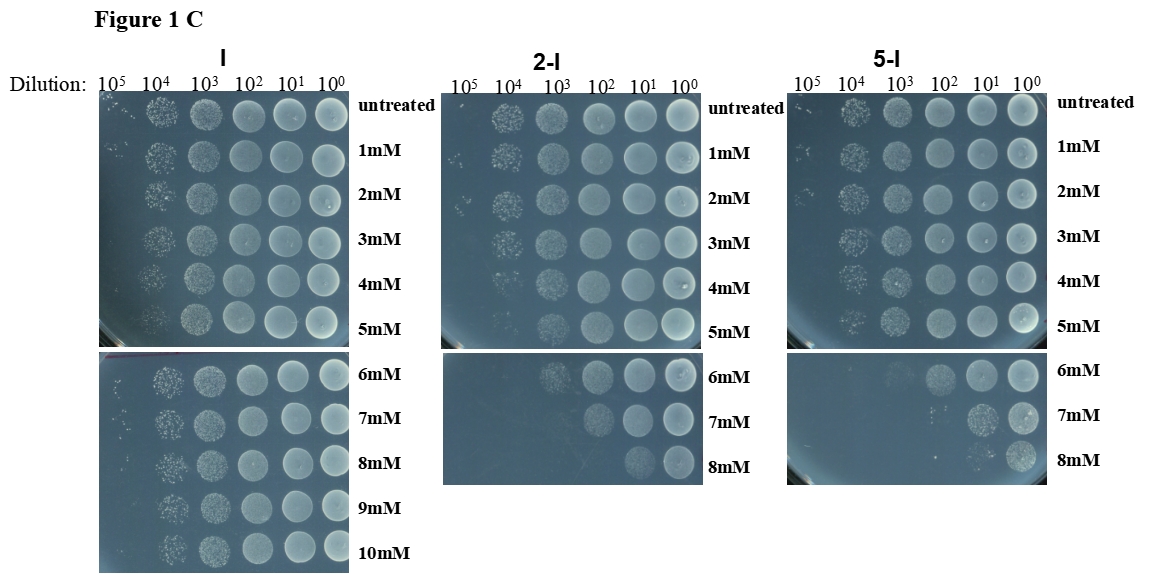


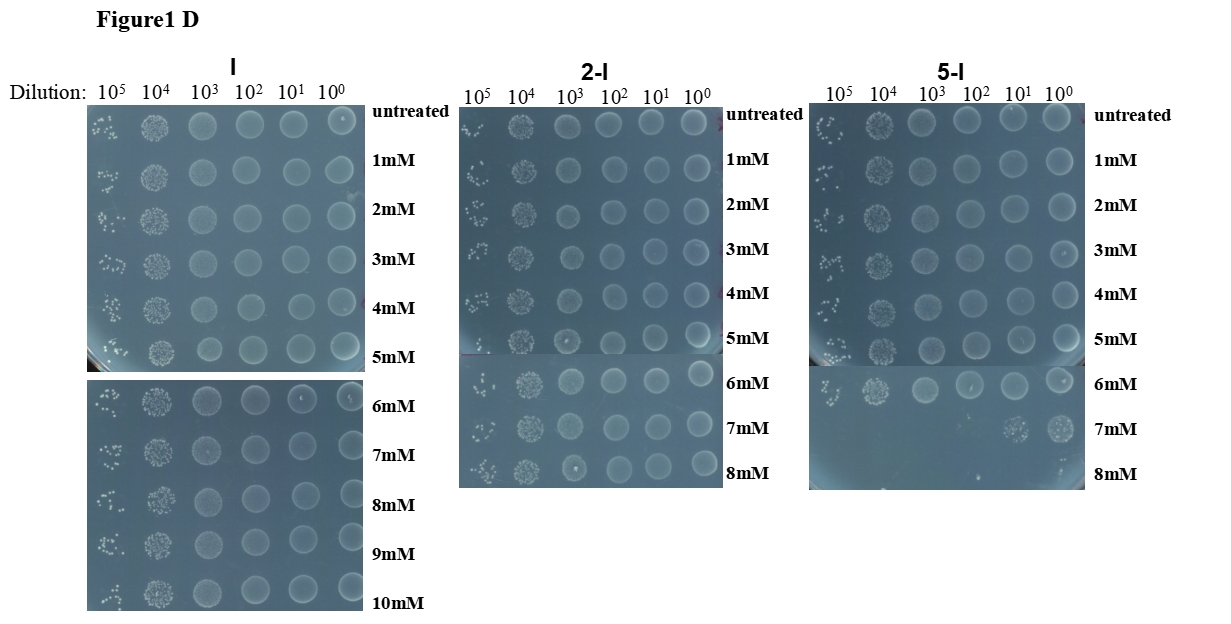


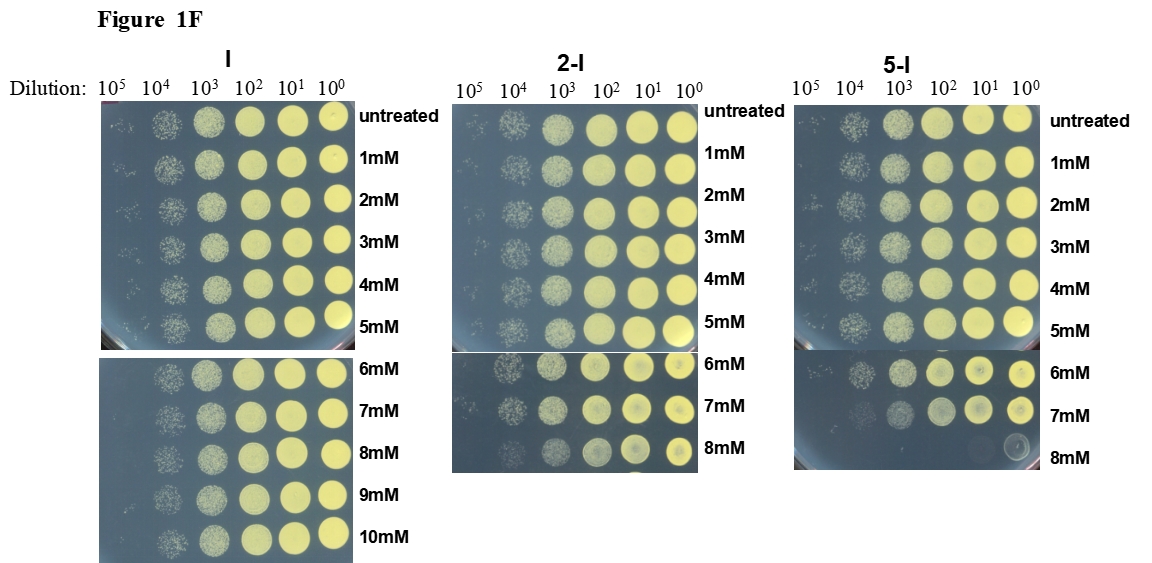


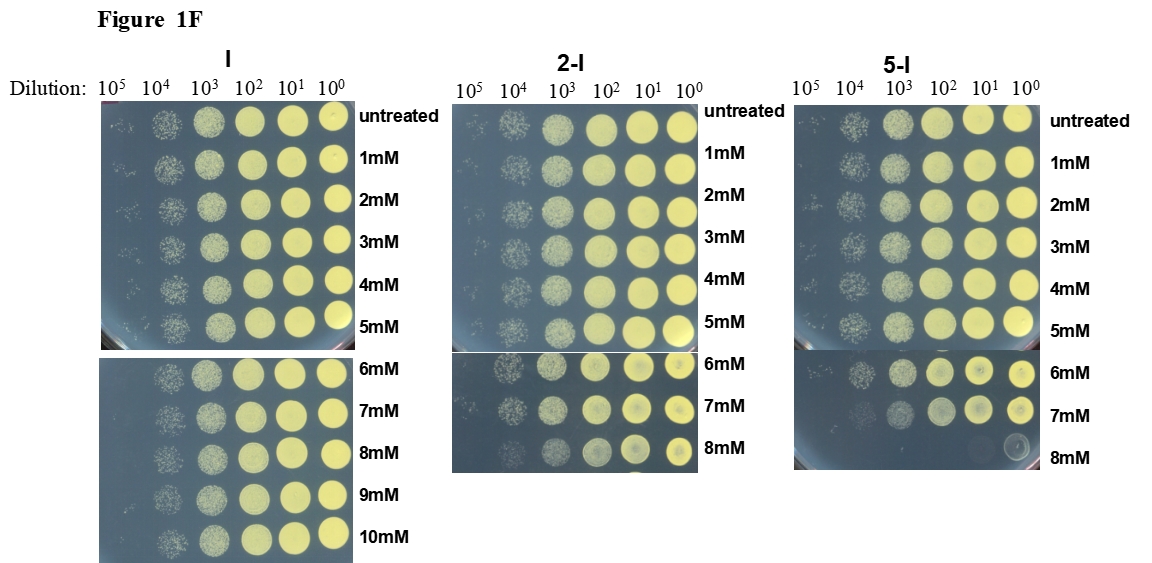


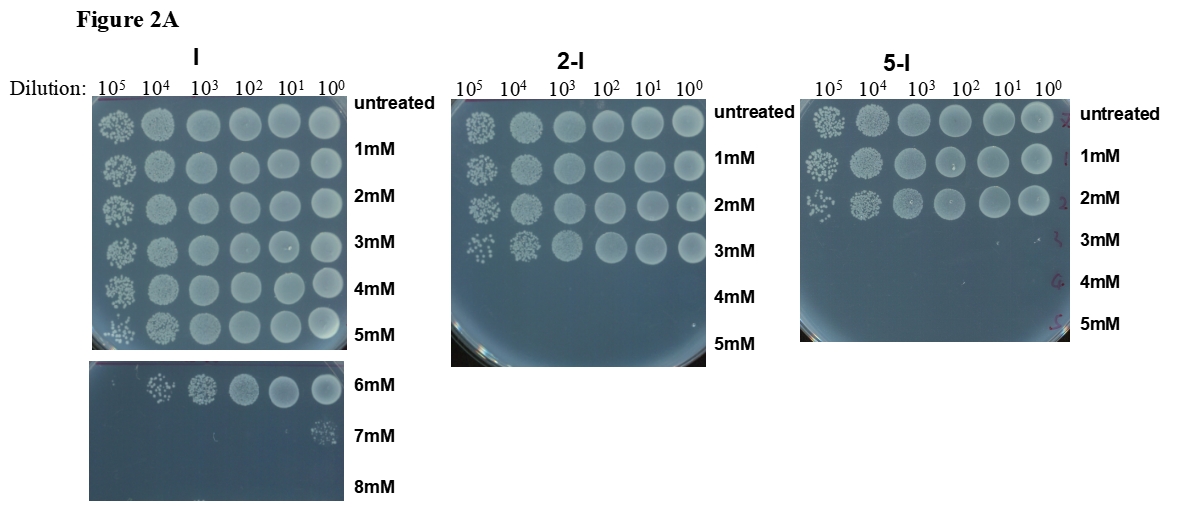


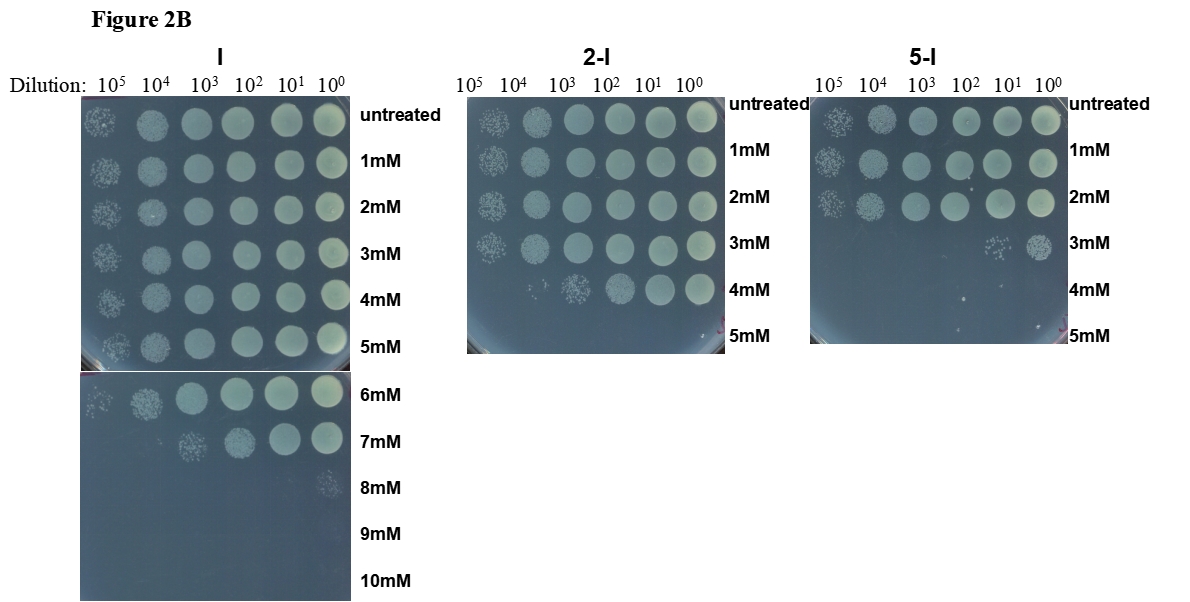


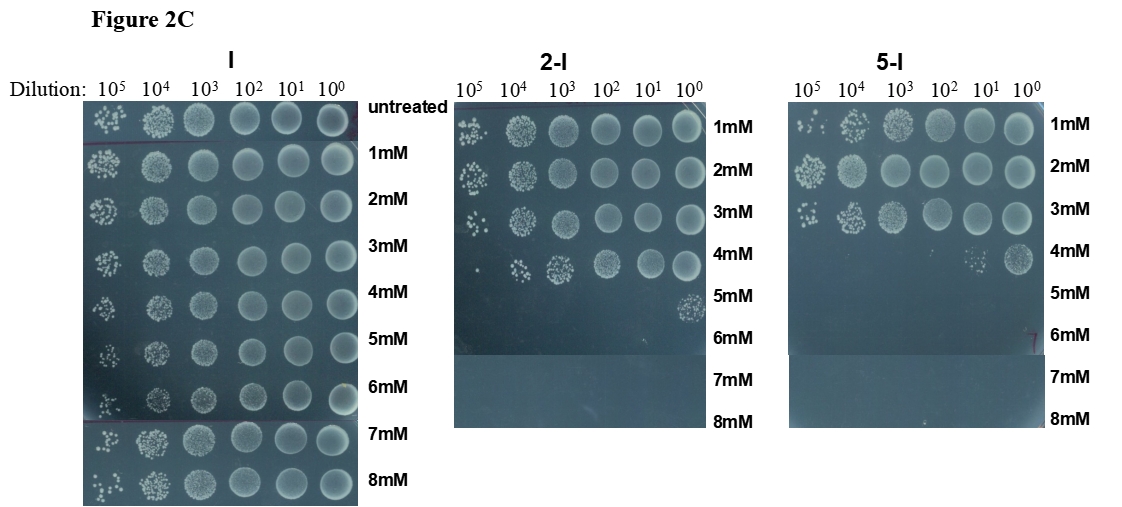


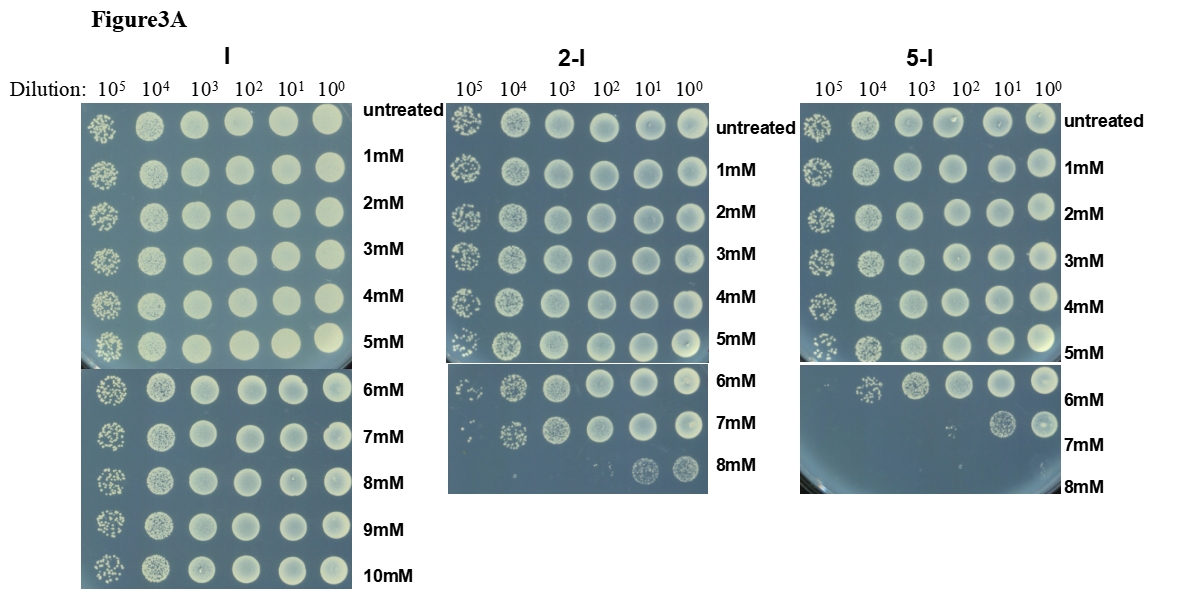


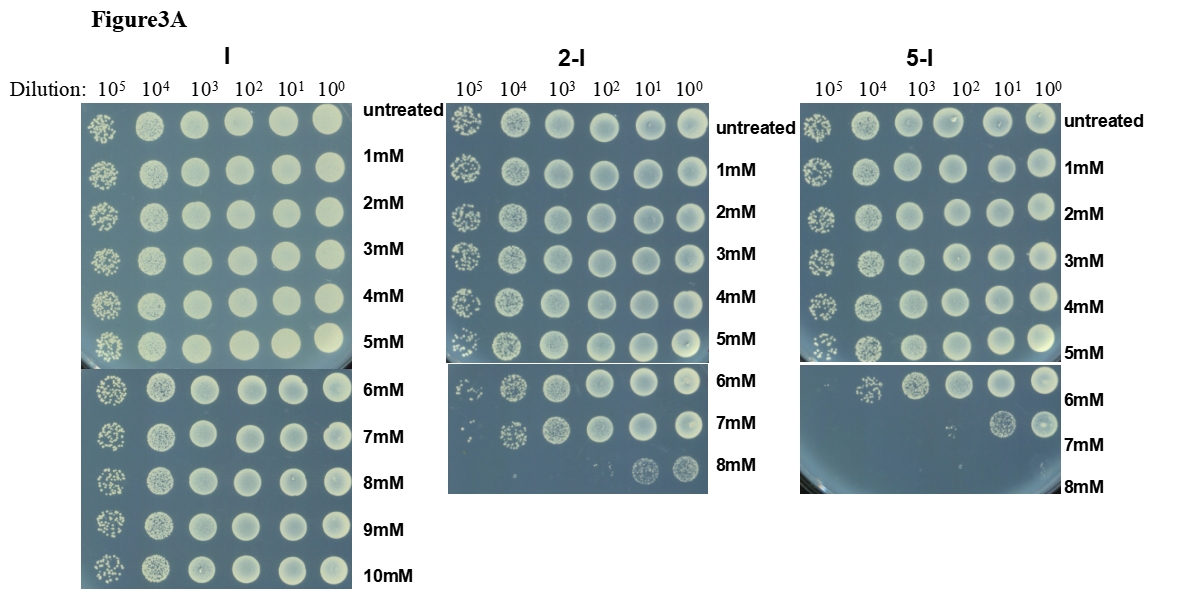


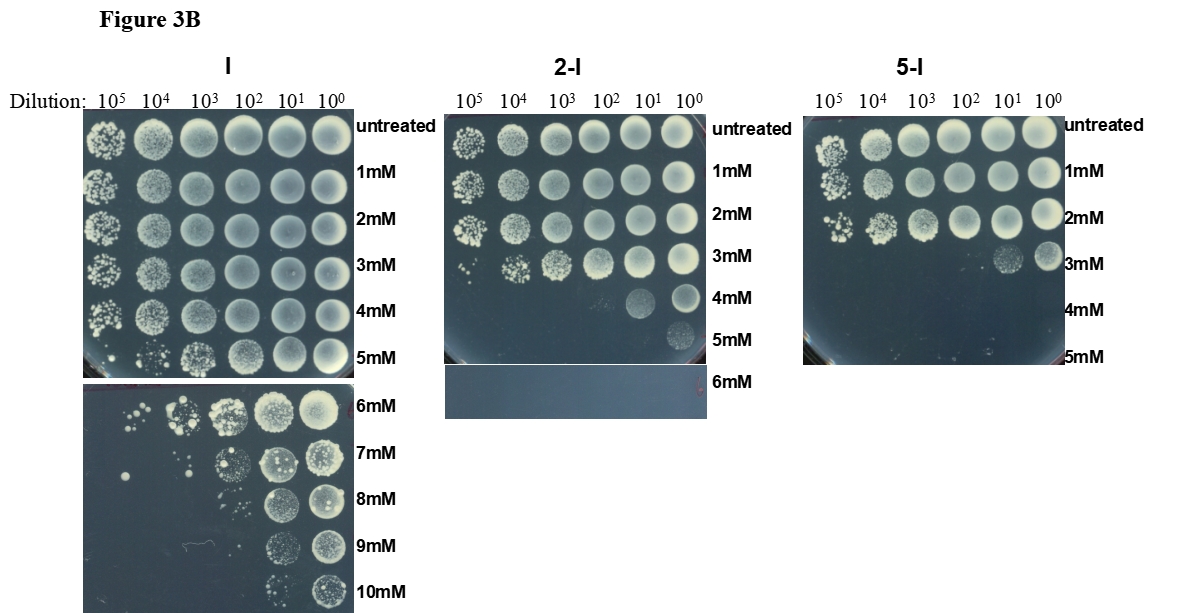


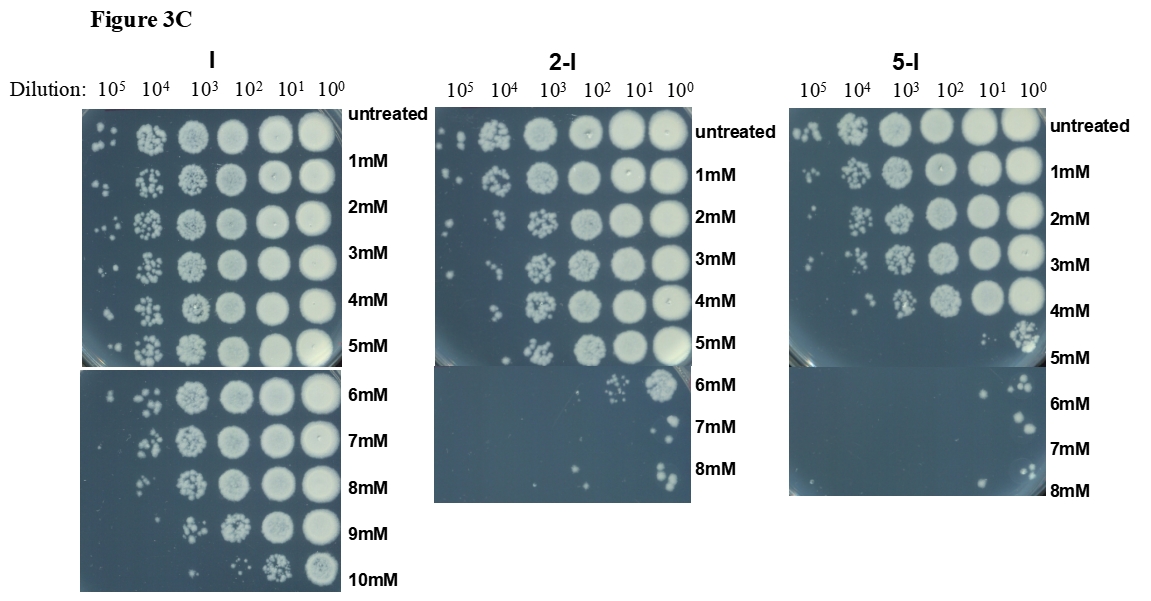


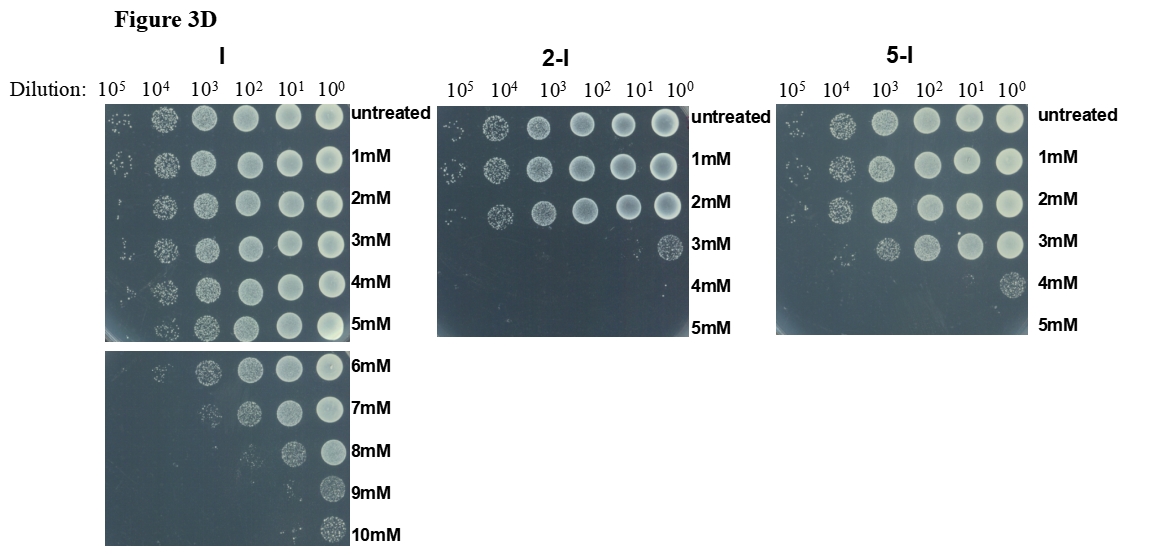


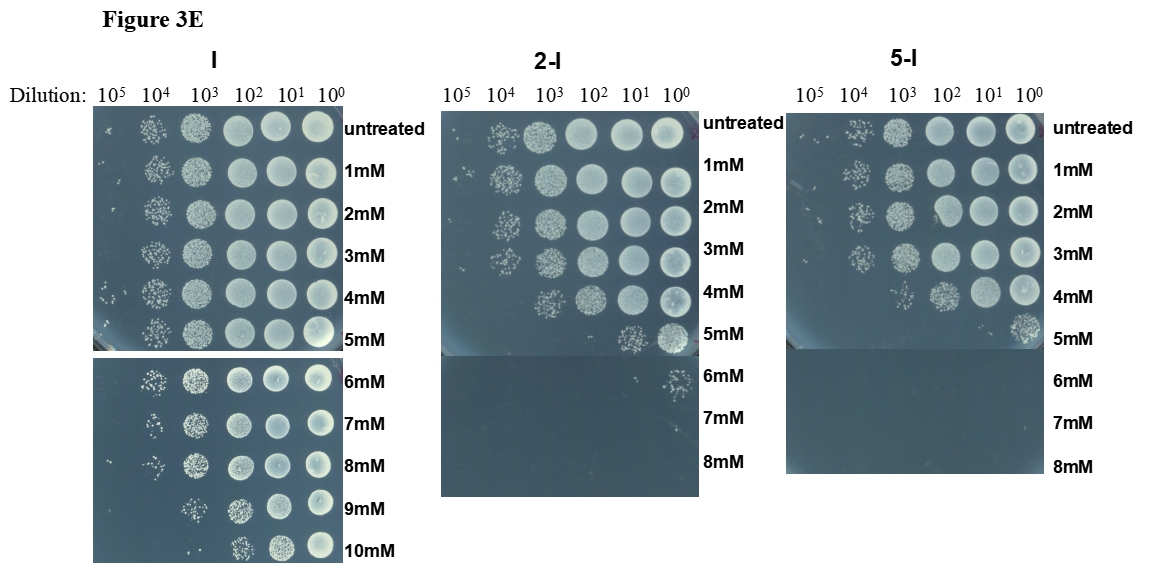


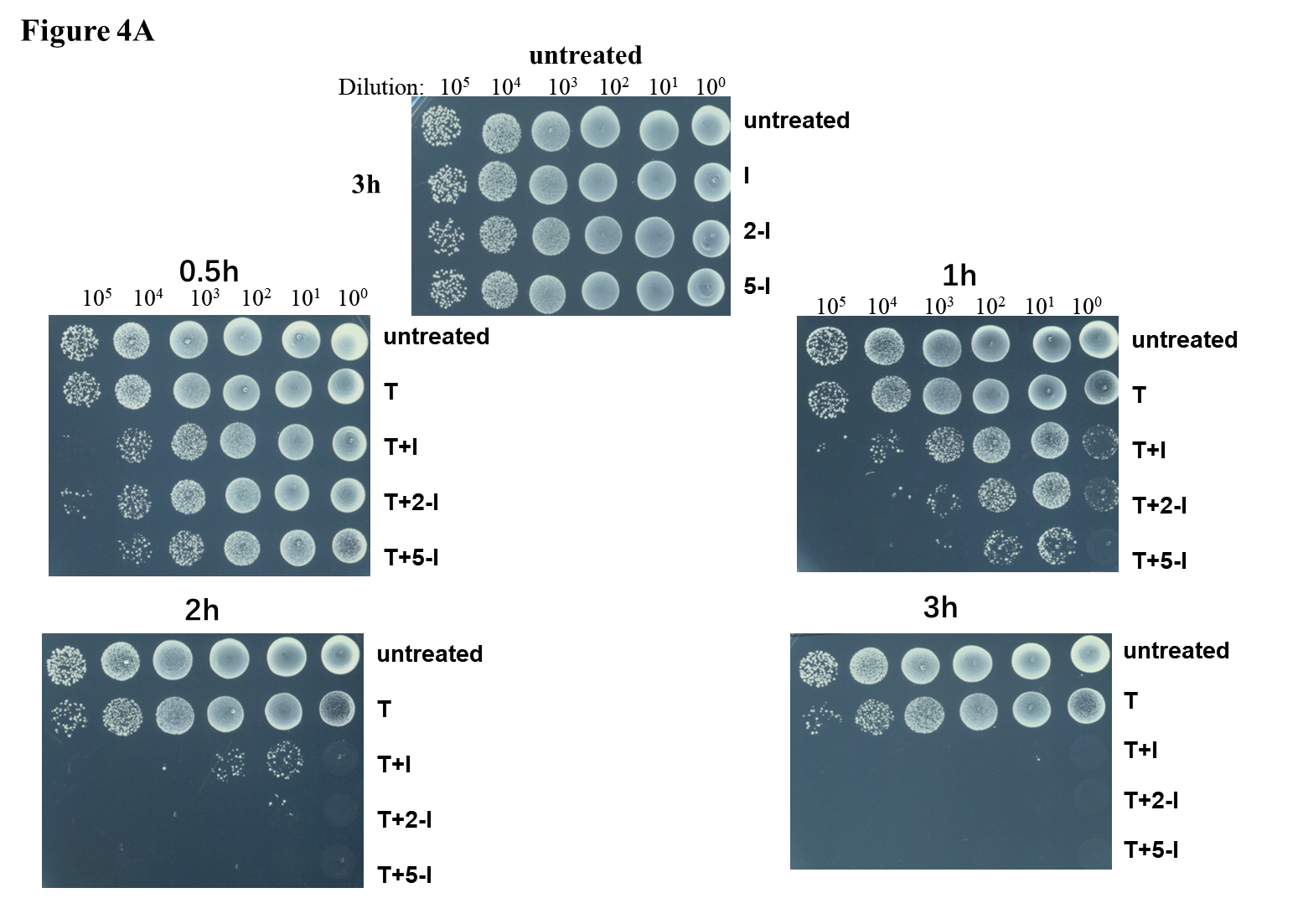


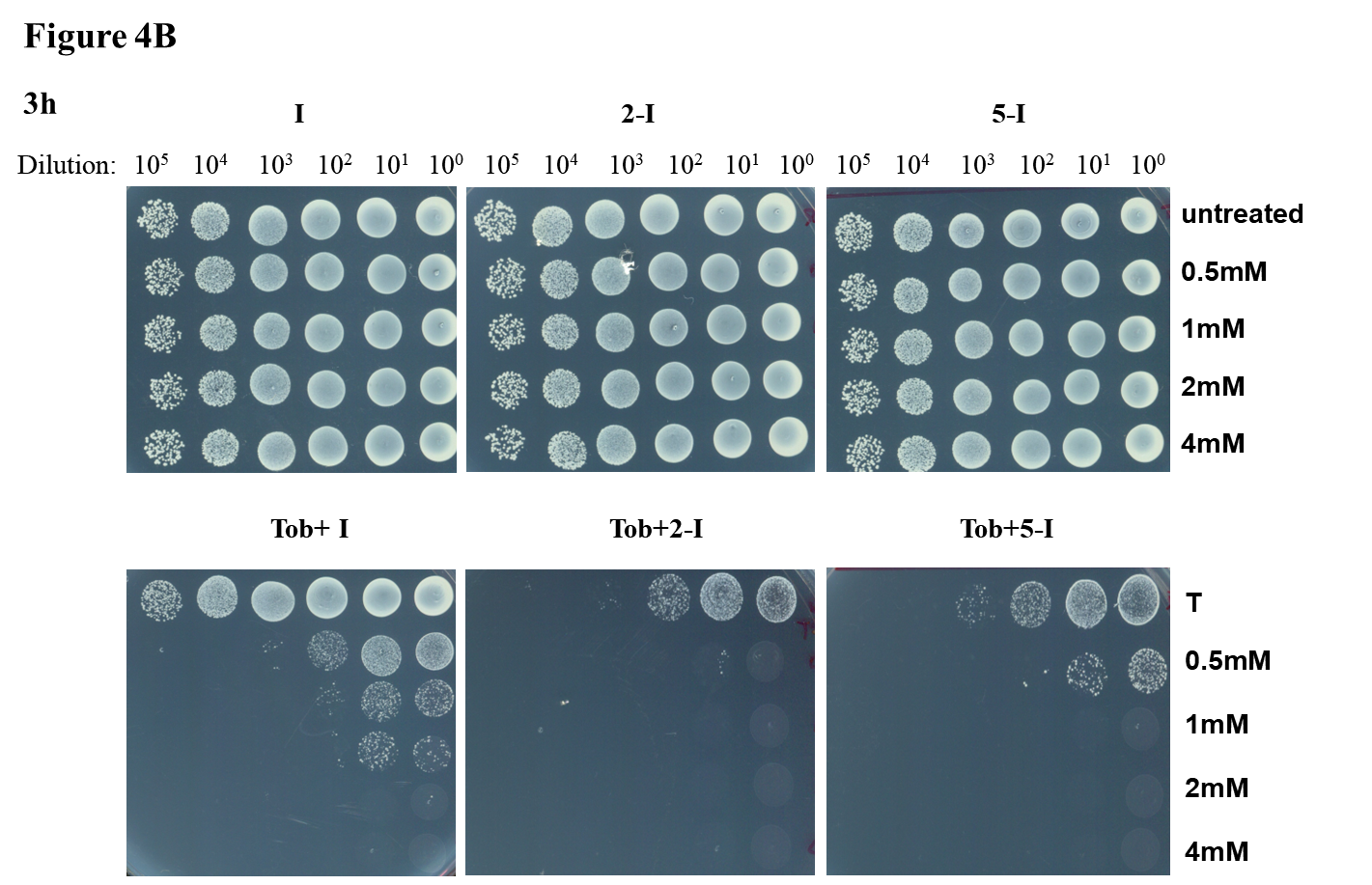


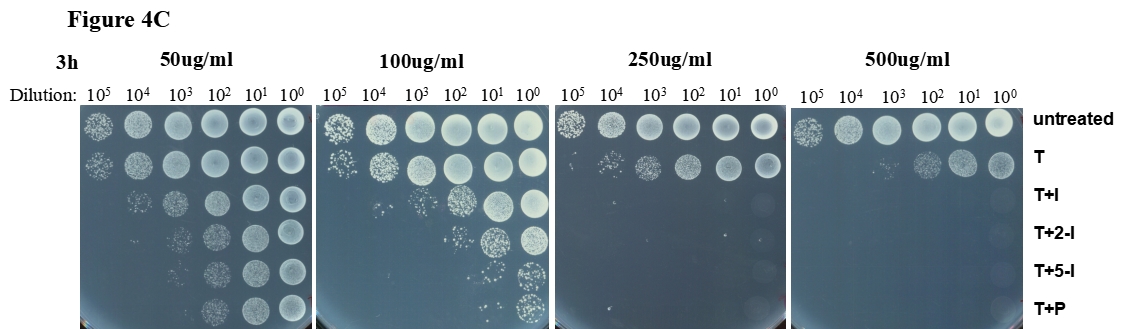


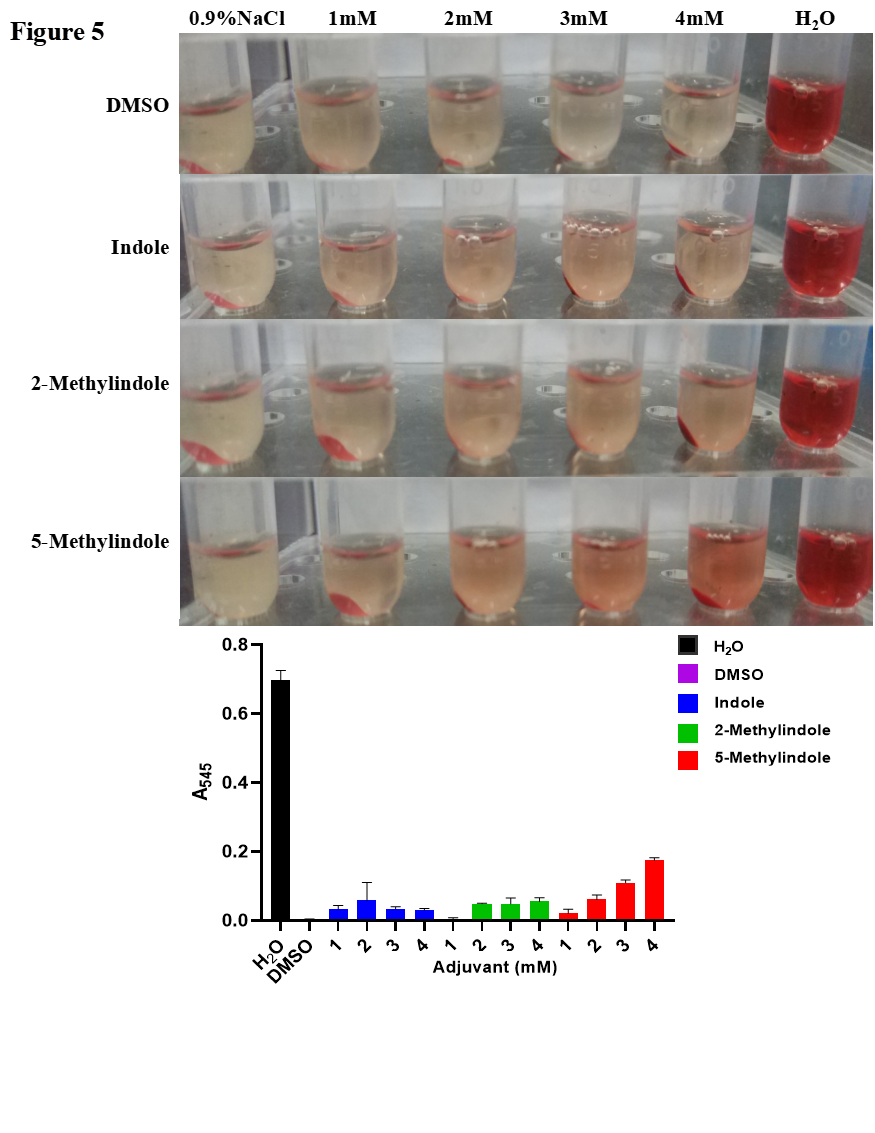


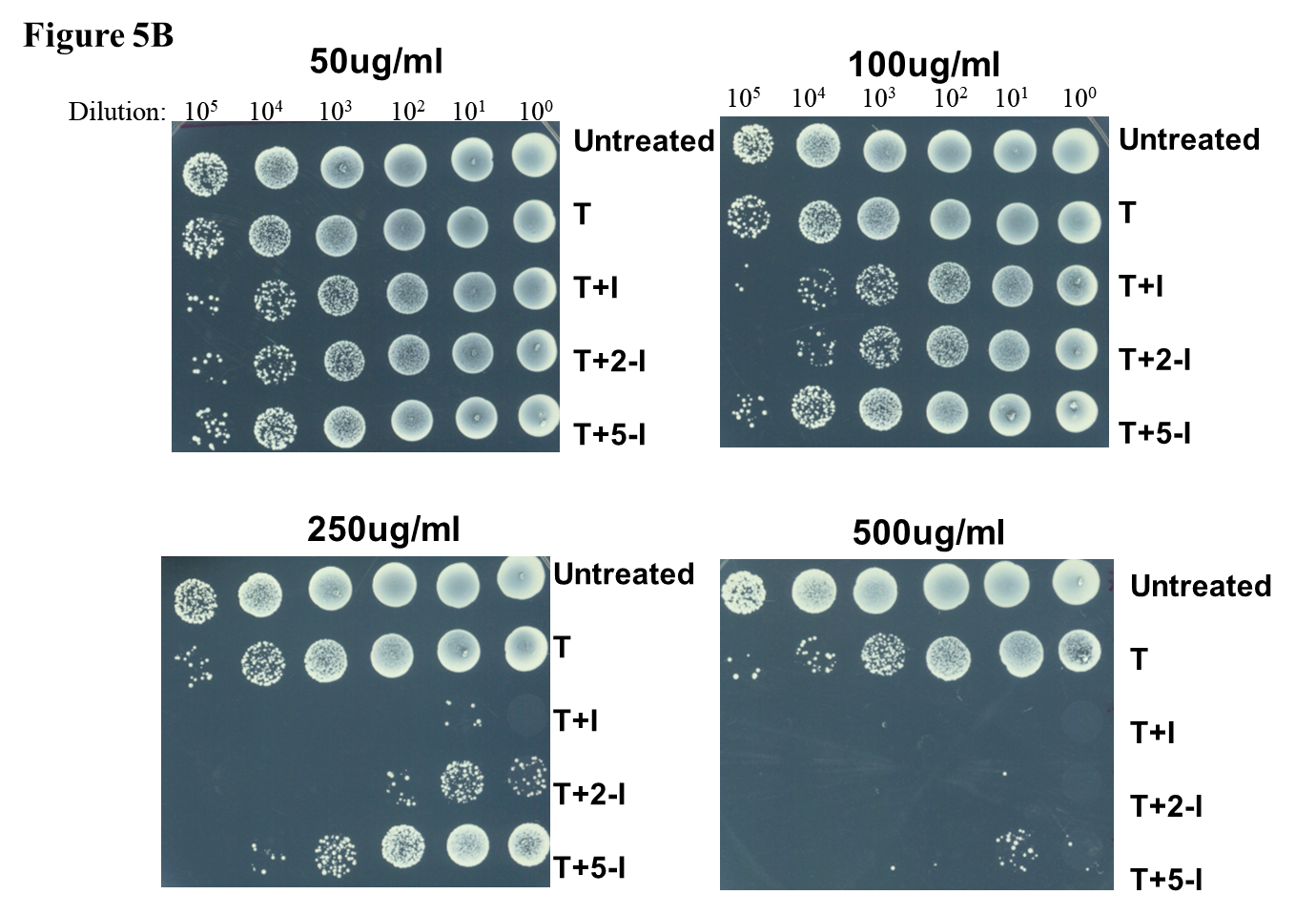


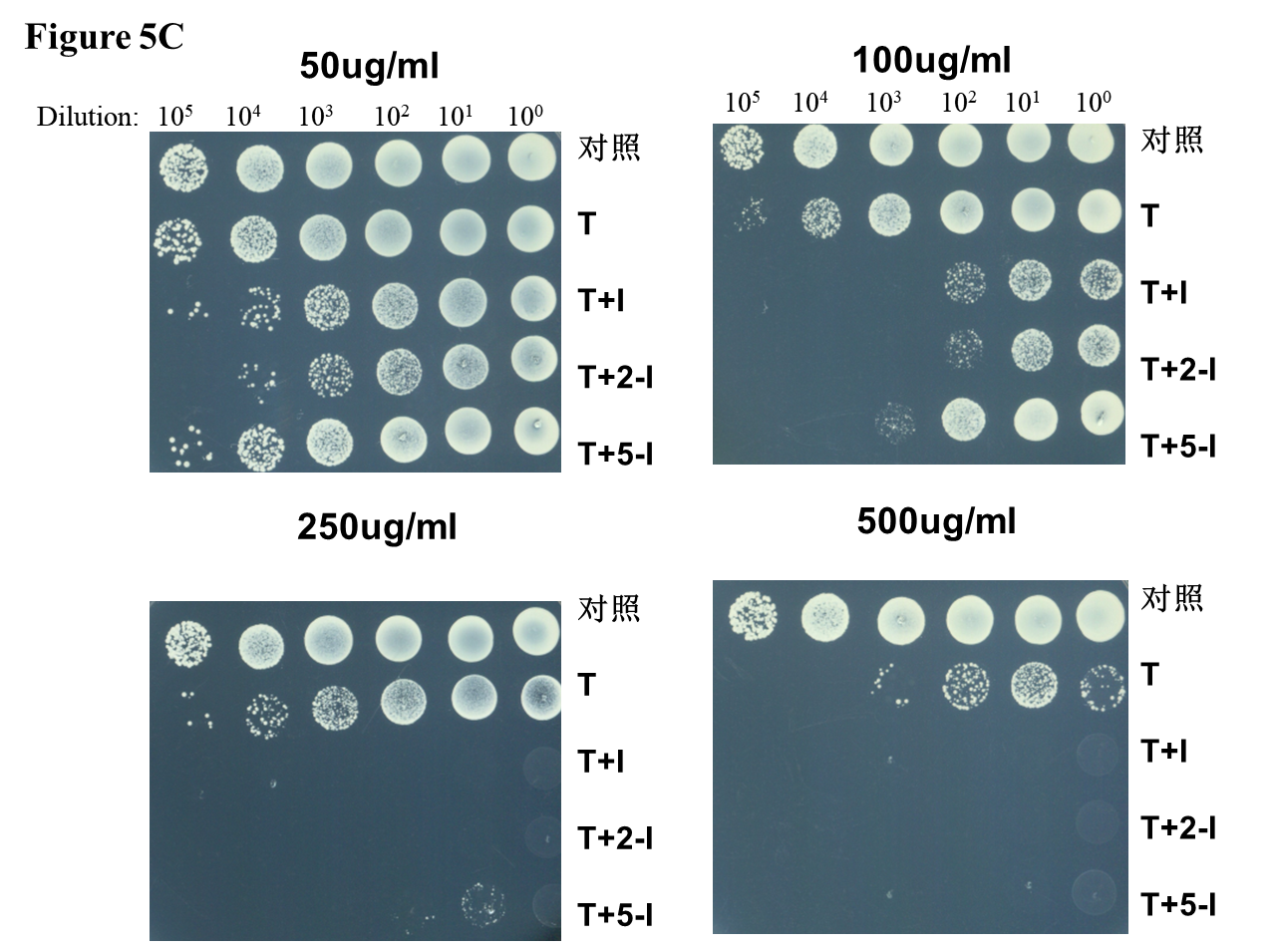

Supplement: Supplemental Information 1 [file peerj-10-14010-s001.docx]

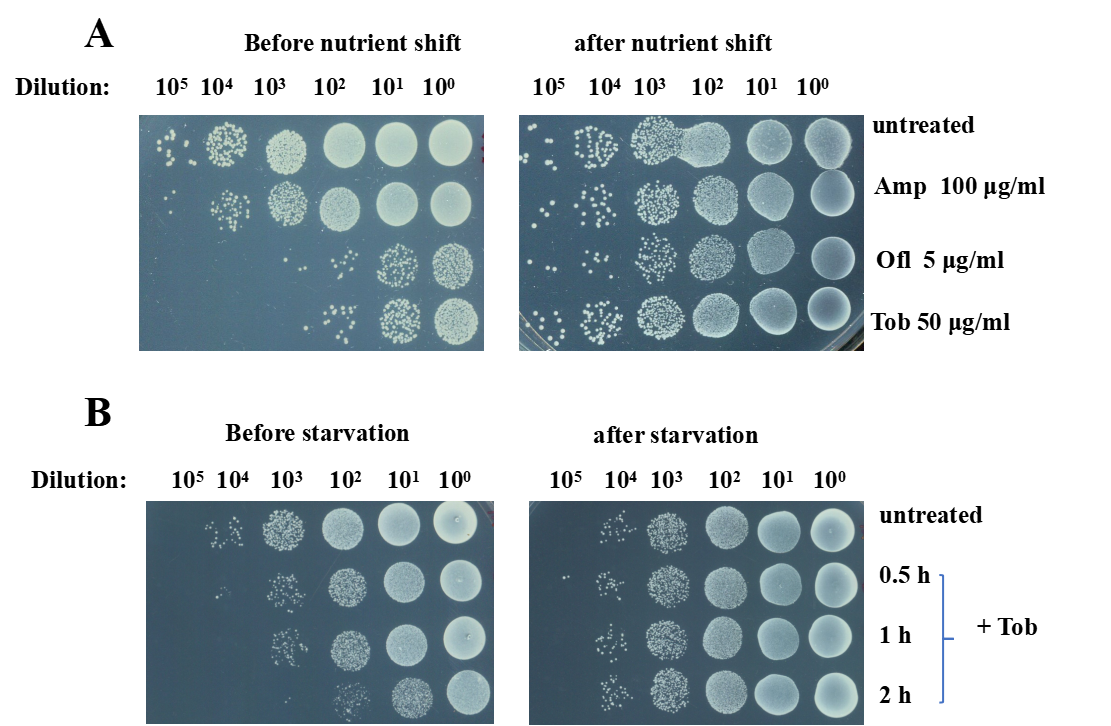

Supplement: Supplemental Information 4 — (A) Survival of S. aureus cells after nutrient shift followed by a 2-h treatment with indicated antibiotics (100 μg/ml ampicillin, 5 μg/ml ofloxacin, and 50 μg/ml tobramycin). (B) Survival of S. aureus cells after starvation followed by 50 μg/ml tobramycin treatment for varying length of time (0.5, 1, or 2 h). [file peerj-10-14010-s004.png]

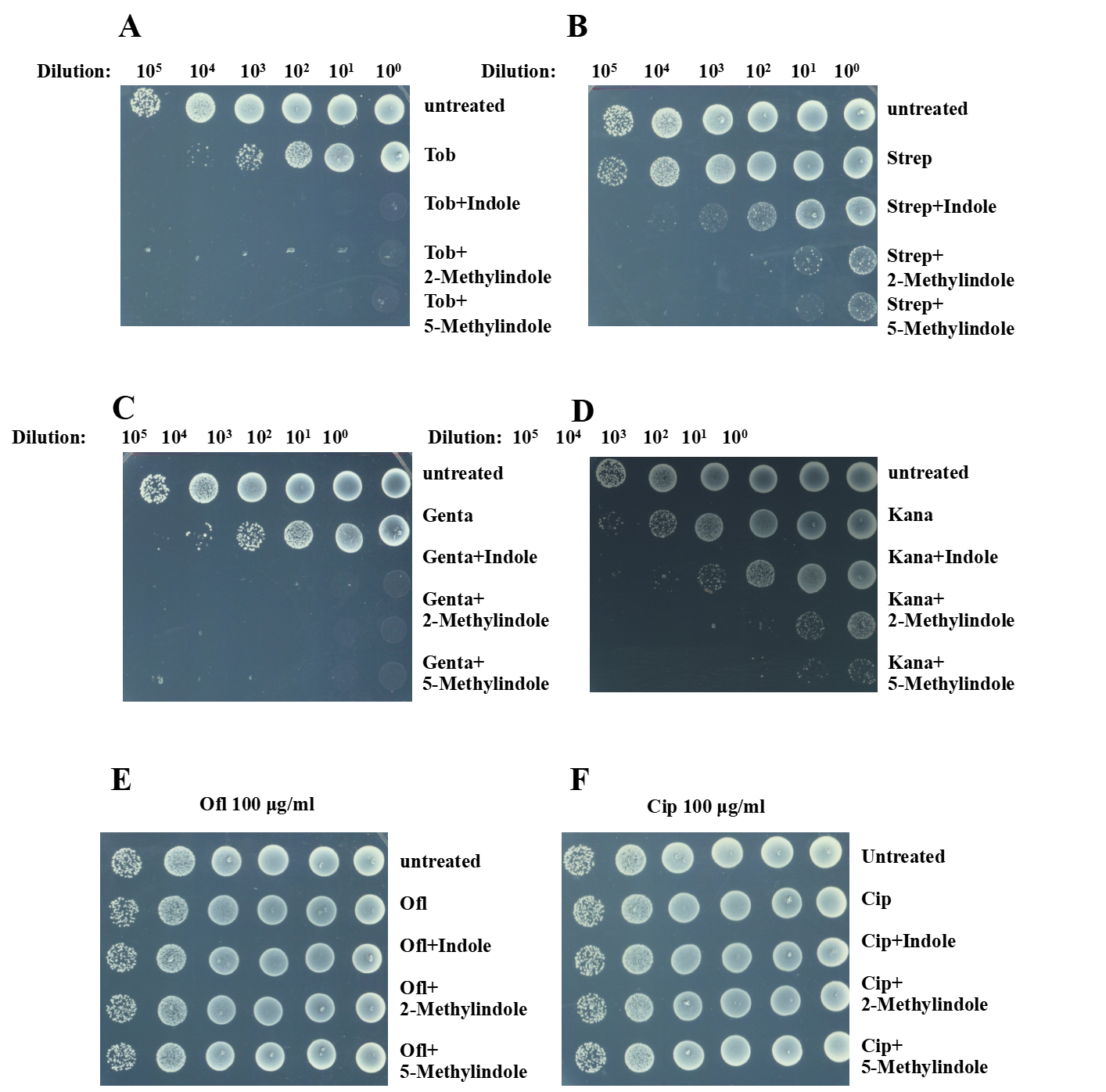

Supplement: Supplemental Information 5 — (A, B, C, D, E, F) Survival of stationary-phase S. aureus cells following a 3-h treatment with 500 μg/ml tobramycin (panel A), 2,000 μg/ml streptomycin (panel B), 500 μg/ml gentamicin (panel C), 1,000 μg/ml kanamycin (panel D), 100 μg/ml ofloxacin (panel E) or 100 μg/ml ciproxacin (panel F) in the absence or presence of 4 mM indole, 2-Methylindole or 5-Methylindole. [file peerj-10-14010-s005.png]

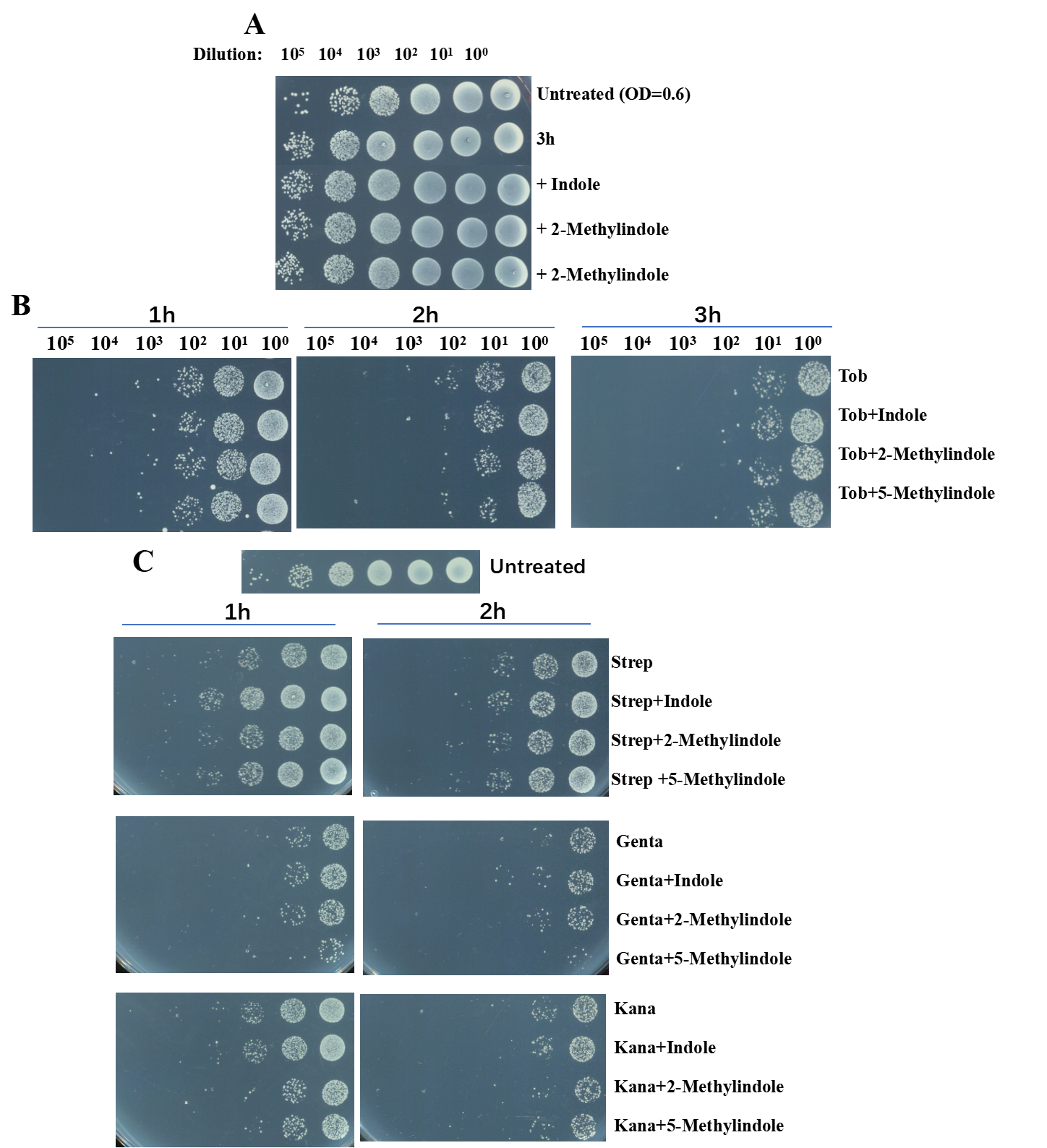

Supplement: Supplemental Information 6 — (A) Survival of exponential-phase S. aureus cells following a 3-h treatment with 1 mM indole, 2-Methylindole or 5-Methylindole. (B) Survival of exponential-phase S. aureus cells following treatments with 25 μg/ml tobramycin plus 1 mM indole, 2-Methylindole or 5-Methylindole for 1, 2 or 3 h. (C) Survival of exponential-phase S. aureus cells following treatments with 100 μg/ml streptomycin, 25 μg/ml gentamicin or 50 μg/ml kanamycin plus 1 mM indole, 2-Methylindole or 5-Methylindole for 1 or 2 h. [file peerj-10-14010-s006.png]

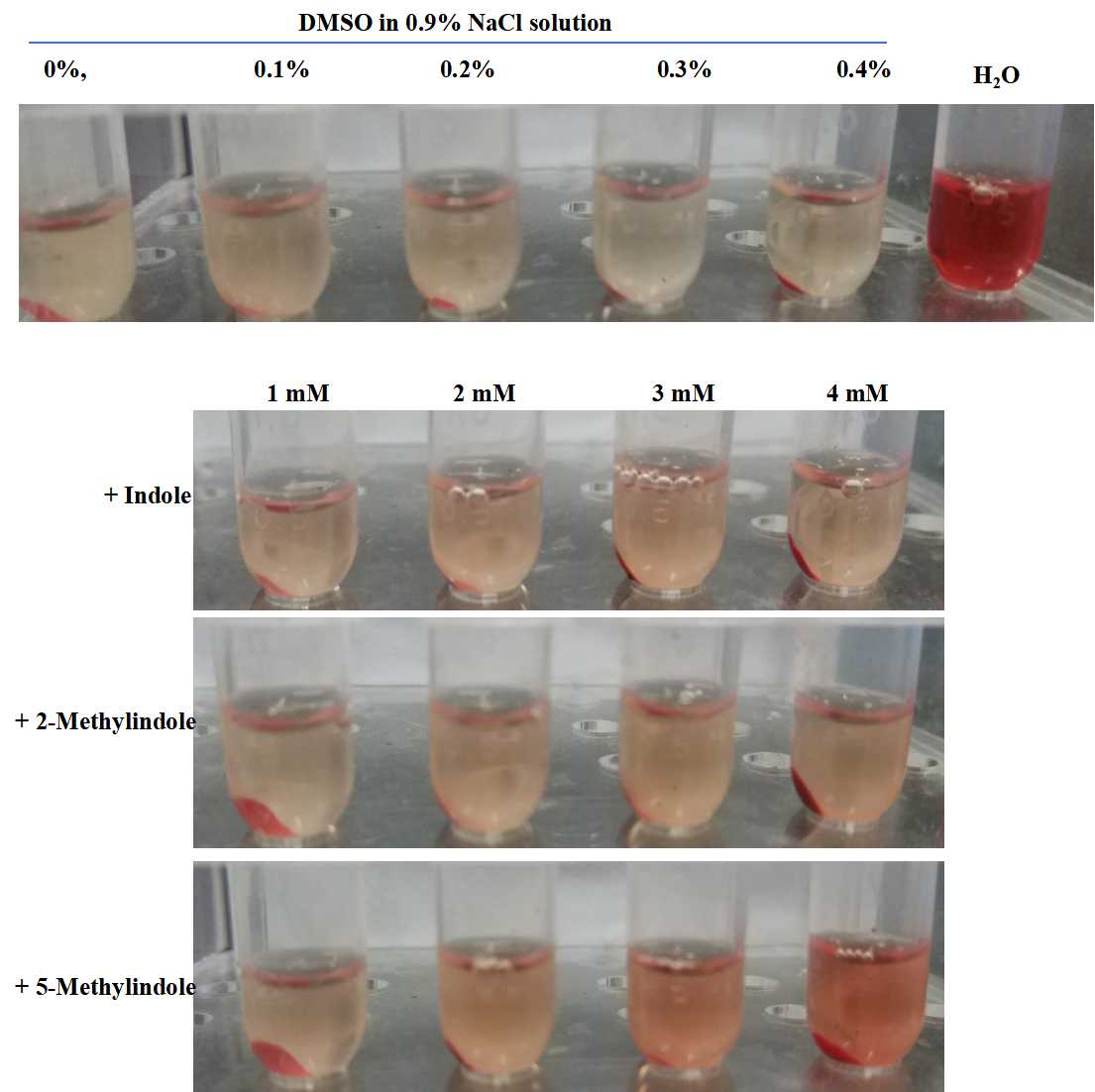

Supplement: Supplemental Information 7 — Upper part: Photos for mouse blood cells dissolved in 0.9% NaCl containing increasing concentrations of DMSO (0, 1, 2, 3 and 4 mM). Cells dissolved in pure water were used as a positive control, i.e., red blood cells were largely lysed in such hypoosmotic solution. Lower part: Photos for mouse blood cells dissolved in 0.9% NaCl solution containing increasing concentration of indole, 2-methylindole or 5-methylindole (0, 1, 2, 3 and 4 mM). [file peerj-10-14010-s007.jpg]

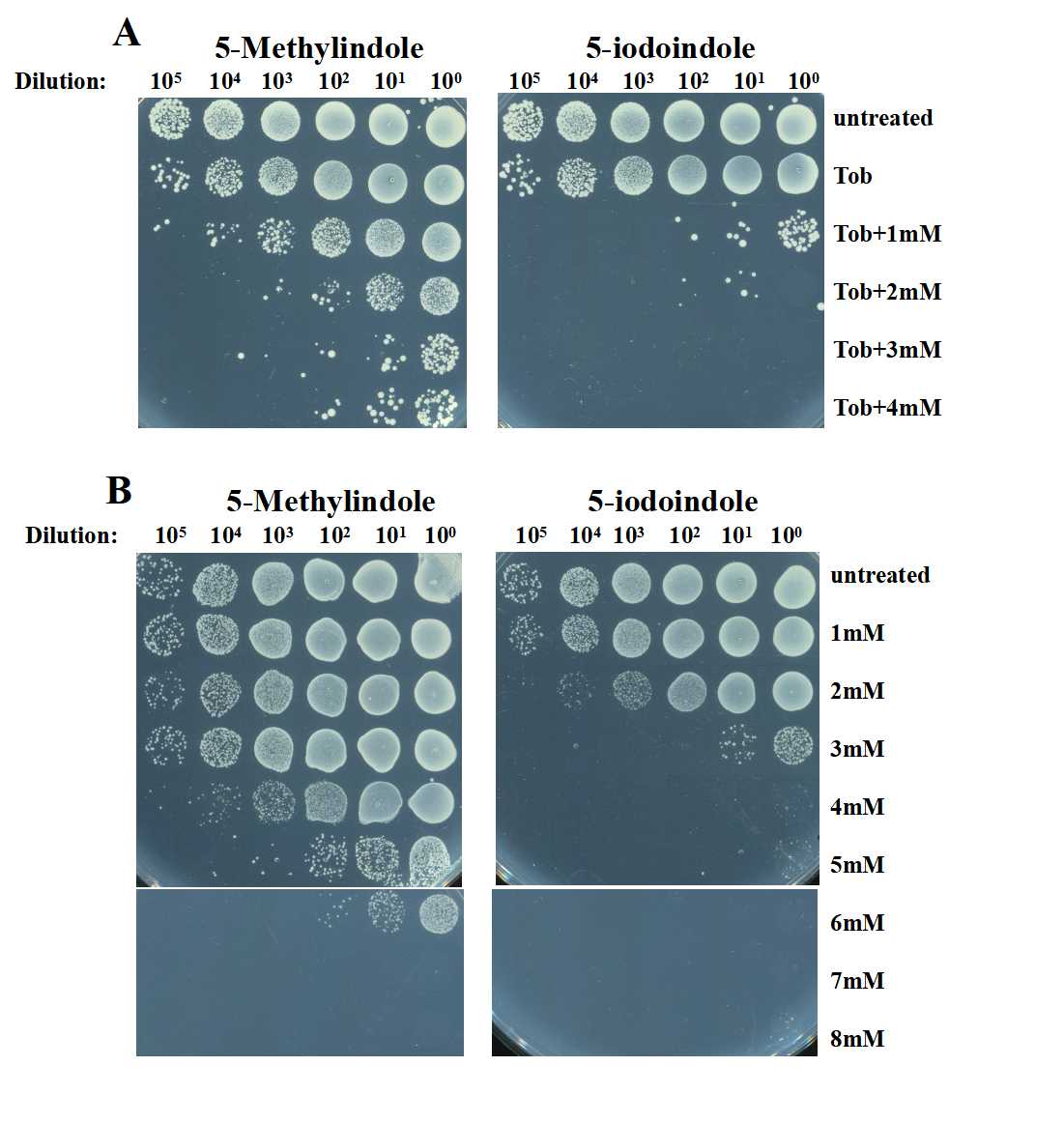

Supplement: Supplemental Information 8 — (A) Survival of stationary-phase S. aureus cells following a 3-h treatment with 250 μg/ml tobramycin plus 5-methylindole or 5-iodoindole (0, 1, 2, 3 and 4 mM). (B) Survival of stationary-phase S. aureus cells following a 3-h treatment with 5-methylindole or 5-iodoindole at increasing concentrations (0, 1, 2, 3, 4, 5, 6, 7 and 8 mM). [file peerj-10-14010-s008.jpg]
