# Supplementary material for: 5-Methylindole kills various bacterial pathogens and potentiates aminoglycoside against methicillin-resistant Staphylococcus aureus
Source: PeerJ. 2022 Sep 14;10:e14010. doi: 10.7717/peerj.14010 (PMC9482361; doi:10.7717/peerj.14010)
Supplement: Supplemental Information 3 [file peerj-10-14010-s003.docx]

| **Bacterial strains** | **Origins** | **Characteristics** |
| --- | --- | --- |
| *Staphylococcus aureus*  *ATCC25923* | A gift from Prof. Luhua Lai at  Peking University | G+ |
| Methicillin-resistant  *Staphylococcus aureus*  ATCC43300 | Hangzhou Binhe Microorganism Reagent Co, Ltd. | G+, multi-drug resistant,  streptomycin-sensitive |
| *Staphylococcus epidermidis*  *CMCC26069* | Hangzhou Binhe Microorganism  Reagent Co, Ltd. | G+,  Streptomycin-resistant |
| *Enterococcus faecalis*  *ATCC29212* | Hangzhou Binhe Microorganism  Reagent Co, Ltd. | G+, multi-drug resistant |
| *Streptococcus pyogenes ATCC19615* | Purchased from Hangzhou  Binhe Microorganism Reagent Co, Ltd. | G+, multi-drug resistant |
| *Micrococcus luteus*  *CMCC28001* | A gift from Dr. Qingeng Huang  at Fujian Normal University | G+, tobramycin- and  kanamycin-resistant |
| *Streptococcus iniae* | Purchased from Hangzhou  Binhe Microorganism Reagent Co, Ltd. | G+, multi-drug resistant |
| *Escherichia coli* BW25113 | the Nara Institute of Science and  Technology (Ikoma, Nara, Japan) | G-, the parent strain  (wild type) for Keio collection |
| *Pseudomonas aeruginosa* PAO1 | A gift from Dr. Zhexian Tian at  Peking University | G-, kanamycin- and  ampicillin-resistant |
| *Shigella flexneri* 24T7T | A gift from Prof. Xiaoyun Liu at  Peking University | G-,  Streptomycin-resistant |
| *Klebsiella pneumoniae*  KP-D367 | A gift from Prof. Xuanxian Peng  at Sun Yat-Sen University | G-, multi-drug resistant |
| *Mycobacterium tuberculosis*  H37Ra | A gift from Prof. Zengyi Chang  at Peking University |  |
